# Heat Stress and Kidney Injury: A Growing Concern Amidst Climate Change

**DOI:** 10.1016/j.xkme.2025.101215

**Published:** 2025-12-15

**Authors:** Safa Y. Mohammed, Abdulqadir J. Nashwan

**Affiliations:** 1 Sinnar University, Sinnar State, Sudan; 2 Nursing and Midwifery Research Department, Hamad Medical Corporation, Doha, Qatar

**Keywords:** Heat stress, kidney injury, climate change, risk factors, dehydration

## Abstract

Rising global temperatures owing to climate change have direct and harmful effects on kidney health, mainly through heat stress and related acute kidney injury. Despite growing clinical evidence, the nephrology community has yet to incorporate environmental stressors into risk models and guidelines fully. This perspective examines the link between heat stress and kidney injury, identifies vulnerable groups, and discusses long-term effects, including the progression of chronic kidney disease. Drawing on epidemiological data, clinical experience, and real-world observations, we advocate the urgent adoption of preventive measures, enhanced clinical training, and climate-aware policies to address the emerging kidney crisis. In addition, this perspective seeks to increase awareness of the risks associated with rising temperatures worldwide, especially in low-income areas, and the risk of heat-related acute kidney injury and kidney disease. It summarizes the concept of heat stress and its impact on kidney health—particularly among high-risk groups, such as those working indoors and outdoors—and the potential mechanisms by which heat stress affects the kidneys. Factors contributing to heat-related kidney disease include dehydration, heat acclimation, age, and other variables. We also outline prevention strategies to lower this risk.

## Introduction

The accelerating pace of climate change is reshaping global health landscapes, with extreme heat emerging as one of the most urgent and pervasive environmental stressors. According to the Intergovernmental Panel on Climate Change, global surface temperatures have risen by ∼1.1 °C above preindustrial levels, and this trend is expected to reach or exceed 1.5 °C within the next 2 decades unless significant mitigation strategies are adopted.^1^ This warming trajectory has led to more frequent, intense, and prolonged heat waves, which now pose a critical threat to human health, especially in vulnerable communities. Studies have documented substantial increases in heat-related morbidity and mortality, with projections showing that vast regions of the globe may experience deadly heat conditions that surpass thresholds for human adaptability within this century.^2^^,^^3^

Although the cardiovascular and respiratory effects of extreme heat have been widely studied, the renal consequences of heat exposure remain underrecognized in both clinical nephrology and public health frameworks. This is because kidneys, owing to their high metabolic demands and dependence on precise perfusion dynamics, are among the most susceptible organs to thermal injury. During periods of extreme heat, physiologic adaptations redirect blood flow from visceral organs, including the kidneys, to the skin surface to facilitate heat dissipation. This redistribution, compounded by dehydration and volume loss, leads to renal hypoperfusion, ischemia, and heightened susceptibility to acute kidney injury (AKI).^4^^,^^5^

Emerging literature has highlighted heat-induced kidney injury as a growing public health challenge. In rural communities across Central America, an epidemic of chronic kidney disease (CKD) of unknown origin—termed Mesoamerican nephropathy—has been linked to recurrent episodes of heat stress and dehydration among agricultural laborers.^6^^,^^7^ Similarly, longitudinal studies in Nicaragua have documented declines in kidney function over a single harvest season among sugarcane cutters exposed to excessive heat, underscoring the cumulative renal toll of occupational exposure.^5^ These findings raise urgent concerns for other regions, such as South Asia and the Middle East, where climate models project sustained periods of extreme heat.^8^

Despite these alarming trends, the renal implications of climate change remain underrepresented in nephrology curricula, clinical guidelines, and preparedness planning. This perspective explores the emerging link between heat stress and kidney injury, offering a detailed overview of the pathophysiologic mechanisms, clinical presentation, at-risk populations, and long-term consequences of heat-induced renal dysfunction. It further underscores the need for integrated clinical, epidemiological, and policy-level responses to address what may become a defining nephrological challenge of the 21st century.

## Understanding Heat Stress and its Clinical Forms

### Thermoregulation

Typically, the human body maintains its core temperature at around 37 °C (98.6 °F) within a very narrow safe range. The body can tolerate cooling (hypothermia) to some extent. But if the temperature increases above 40.5 °C (104.9 °F), the risk of multiple organ failure increases rapidly.^1^ The body maintains a balance between heat generated from metabolic processes and heat absorbed from the environment using various methods of heat dissipation. These central, integrated pathways for heat loss are regulated by the body’s central thermostat in the brain. The thermostat center sends signals through the autonomic nervous system, triggering processes of vasodilation of cutaneous blood vessels in the skin and sweating (diaphoresis) to help the body release heat.^1^

**Figure 1:**
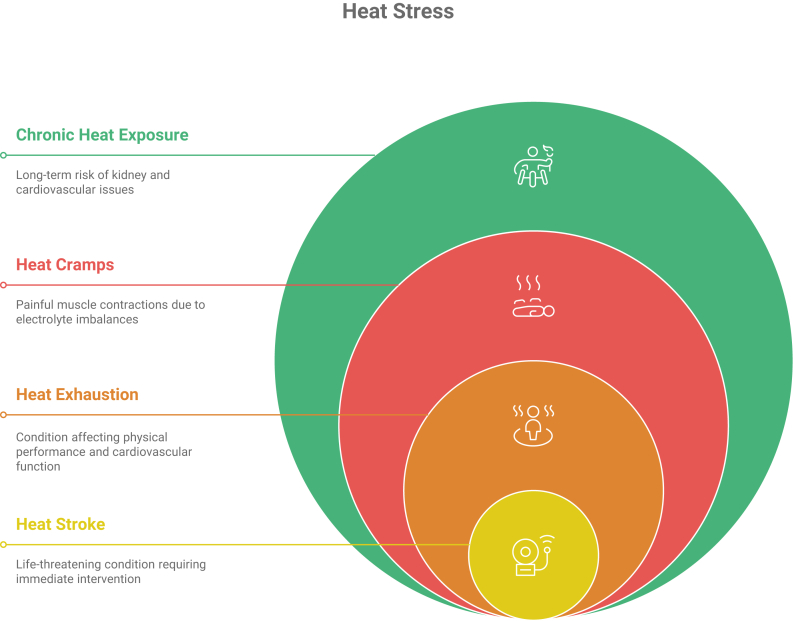
Heat-related illnesses.

In prolonged exposure to an extreme heat environment, the body’s ability to thermoregulate itself will be overwhelmed, leading to hyperthermia, which is an increase in core body temperature.^2^ This condition is called heat stress. It can exacerbate pre-existing health issues and lead to heat-related illnesses (HRIs), including heat exhaustion, heat cramps, and heat stress.^3^^,^^4^ Heat stress is the broad term for HRIs, which range from mild muscle cramps to the life-threatening condition of heat stroke (Fig 1).

### Populations at Elevated Risk

Heat stress broadly arises in vulnerable groups, such as older adults, children, and individuals with chronic conditions, including diabetes and hypertension, following passive exposure to elevated ambient temperatures.^9^ Many factors further compound vulnerability, such as occupational and environmental factors that affect specific groups at risk, including outdoor laborers (eg, farmers and construction workers), military recruits, and athletes who often operate in high-heat environments without adequate hydration or rest, in addition to indoor workers who are exposed to artificial heat sources.^10^ In urban areas, the “heat island” effect raises ambient temperatures, thereby increasing the risk among socially disadvantaged populations.^3^^,^^11^

Despite differences in presentation, all heat stress forms ultimately converge on thermoregulatory failure, resulting in systemic inflammation, cardiovascular strain, and renal hypoperfusion.^11^

**Figure 2:**
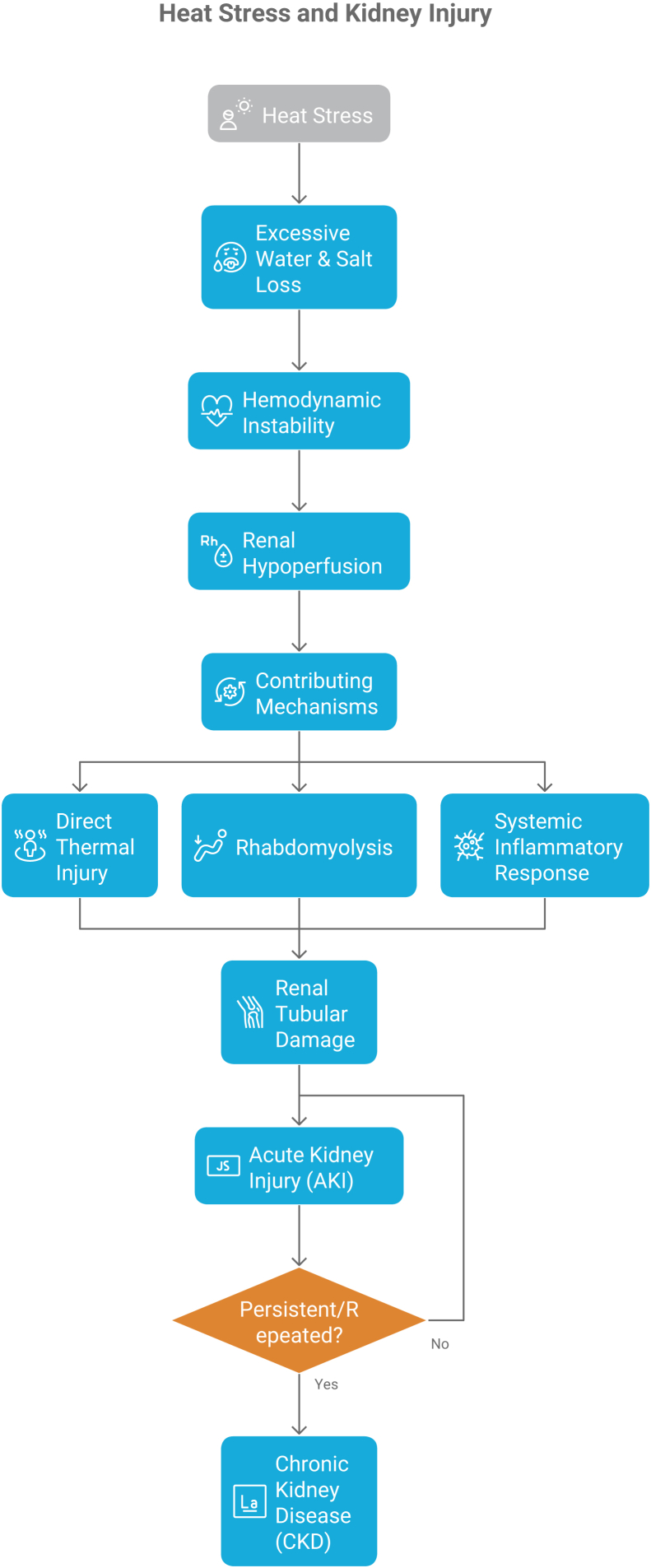
Pathophysiologic cascade from heat stress to kidney injury.

Figure 2 identifies groups including the elderly, young children, individuals with chronic illnesses, outdoor workers, and residents in low-income urban areas as most vulnerable to heat-related kidney failure.

### Pathophysiology of Heat-Related Kidney Injury

The renal effects of heat stress are multifactorial and mediated by hyperosmolarity, vasopressin activation, an increased urinary concentration, and inflammation-toxic mechanisms. During thermal stress, the body redistributes blood from the viscera to the skin to facilitate heat dissipation, reducing renal blood flow.^12^ Concurrent dehydration intensifies intravascular volume depletion and impairs renal perfusion, making the kidneys highly susceptible to ischemic injury and progressing to CKD.^13^^,^^14^

### Heat Stress and Rhabdomyolysis

In the setting of heat stress, rhabdomyolysis is a common occurrence, leading to the release of myoglobin into the bloodstream. Once filtered by the kidneys, myoglobin can accumulate within the renal tubules, where it may cause obstruction and induce direct oxidative toxicity.^12^^,^^15^

### Systemic Kidney Inflammation and Gut Permeability

In situations of heat stress, inflammatory cytokines are released into the bloodstream, leading to endothelial dysfunction, tubular apoptosis, and capillary leaks in the kidneys. These cytokines, such as interleukin 6 and tumor necrosis factor α,^14^ play a crucial role in this process. In addition, there is an increase in gut permeability leading to the release of bacterial endotoxins into circulation, which is called the “inflammatory-toxic” mechanism. This mechanism leads to multiorgan failure and neurological symptoms in exertional heat stress, or more localized damage in heat-related kidney injury.^12^^,^^14^

### Uric Acid Buildup

Dehydration and heat stress contribute to uric acid buildup and kidney illness, although they concentrate uric acid in the urine and increase crystal formation. The kidneys should usually filter these crystals, but owing to heat stress and dehydration, they deposit in the kidney tissues, triggering inflammation and damaging the renal tubules.^12^^,^^15^

### Renin-Angiotensin-Aldosterone System

Prolonged exposure to extreme heat and physical exertion leads to volume depletion, triggering activation of the renin-angiotensin-aldosterone system and vasopressin pathways as compensatory mechanisms to maintain blood pressure and water balance.^16^ However, these hormonal responses lead to renal sodium and water reabsorption at the expense of urinary potassium loss, predisposing workers to hypokalemia. In addition, hypokalemia and hypomagnesemia have been associated with increased risk of incident kidney injury.^12^

### Vasopressin-Induced Kidney Injury

It is well established that vasopressin plays a crucial role in reabsorbing water in the kidneys, thereby maintaining the body’s water balance. In heat stress, the body responds to dehydration by elevating the level of vasopressin, which induces glomerular hyperfiltration and albuminuria. These can damage the kidney filtration units. Furthermore, dehydration activates fructose metabolism and generates an oxidative stressor in the blood, leading to renal tubular injury.^9^^,^^12^

Repeated subclinical ischemic injury from dehydration, combined with detrimental effects, accelerates renal fibrosis and vascular rarefaction, leading to heat stress--induced CKD.^17^, ^18^, ^19^

Up to one-third of the patients who are hospitalized owing to heat stress develop AKI, particularly when diagnosis and treatment are delayed.^16^ Therefore, early laboratory tests to detect heat-related nephropathy contribute to reducing the rate of hospitalization. A diagnostic evaluation should include an assessment of core temperature, serum creatinine, blood urea nitrogen, uric acid, and urine studies. Management primarily focuses on rapid cooling, intravenous fluid resuscitation, and kidney replacement therapy in severe cases.^14^^,^^15^

**Figure 3:**
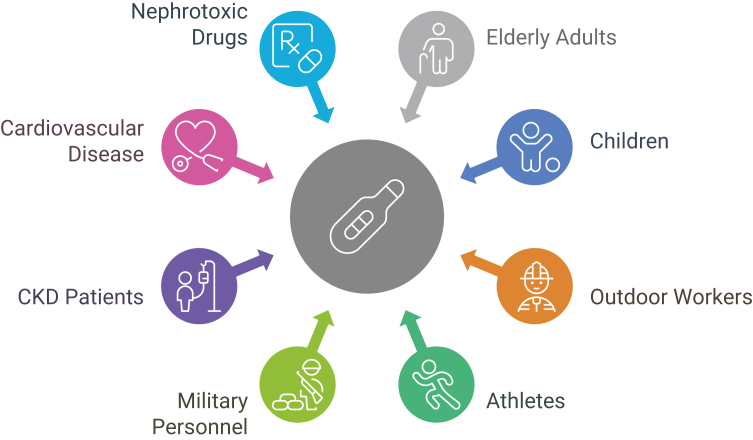
High-risk populations for heat-induced kidney injury. Abbreviation: CKD, chronic kidney disease.

Figure 3 illustrates the path from environmental heat exposure to AKI, including dehydration, hypoperfusion, inflammation, oxidative stress, and myoglobin-mediated tubular toxicity.

### From Acute Injury to Chronic Disease

The implications of heat stress--induced AKI may extend beyond immediate hospitalization. A growing body of evidence links repeated episodes of AKI to incomplete renal recovery and elevated risk of CKD and kidney failure.^17^ Histological studies reveal interstitial fibrosis, tubular atrophy, and glomerulosclerosis following thermal injury to the kidneys. These changes likely reflect a maladaptive repair process perpetuated by ongoing inflammation and mitochondrial dysfunction.^5^^,^^14^

Given the global burden of CKD—estimated to affect over 800 million people worldwide—the contribution of heat-induced AKI represents a potentially modifiable risk factor, especially in tropical and subtropical regions where heat waves are intensifying.^17^

## Factors Affecting HRI

HRIs are influenced by both environmental and individual determinants that are no less important than pathogenic factors in affecting body temperature and the cooling mechanism.^2^^,^^3^

A high ambient temperature and humidity represent critical environmental determinants of heat stress, as they impair the body’s thermoregulatory capacity by limiting sweat evaporation, the primary physiologic cooling mechanism. In addition, radiant heat, whether from direct solar exposure or from contact with hot surfaces such as ovens, pipes, and industrial equipment, as well as clothing and personal protective equipment that trap heat, can hinder the effective dissipation of body heat. Moreover, poor air circulation, particularly in indoor or occupational settings, exacerbates these risks by creating stagnant conditions that not only restrict cooling but also may promote the accumulation of airborne pollutants, including mold and dust. Therefore, monitoring the humidity level and checking for the safety of workplace air are just as important as monitoring the temperature to mitigate the risk of HRI.^2^^,^^3^

It is also known that air pollution from allergens, chemicals, and particulates contributes to the emergence of lung and kidney diseases. Particulates generated from the combustion of coal, gasoline, and diesel fuels represent a significant component of air pollution and are strongly linked to adverse health outcomes, particularly in the kidneys. In addition, other environmental pollutants, such as nitrogen dioxide (NO_2_), carbon monoxide (CO), and toxic heavy metals, including mercury (Hg) and lead (Pb), are a combination of hazardous gases that adversely affect the body. Furthermore, it can lead to systemic kidney dysfunction.^20^^,^^21^

Furthermore, an intense workload in hot weather can lead to AKI and eventually CKD owing to dehydration and the inflammatory process that occurs.^13^

Age is one of the individual factors that influence the risk of HRI. It is known that older adults and young children are a more susceptible group.^22^ Furthermore, young people in our current era have become more susceptible to HRI owing to their involvement in hard work in hot weather, lack of drinking water, and long working hours.^3^

Dehydration is also a significant risk factor contributing to HRI. Insufficient fluid intake during heat exposure impairs the body’s ability to cool down and stimulates the pathogenesis of nephropathy.^3^

Another significant factor contributing to HRI is individual medical conditions, including cardiovascular disease, diabetes, and infectious diseases. They make the kidney system less able to tolerate high ambient temperatures and are easily impaired.

Moreover, there is widespread use of some drugs such as nonsteroidal anti-inflammatory drugs, antibiotics, metformin, sodium-glucose cotransporter 2 inhibitors, and diuretics, which have been shown to contribute to drug-induced nephropathy.

Statistics have shown that kidney toxicity resulting from the repeated use of medications represents 20% of all other causes of kidney toxicity. Besides, studies have shown that drug-induced kidney toxicity is one of the primary causes of kidney disease, along with the common cause, infection.^23^

These drugs may involve direct tubular injury leading to acute tubular necrosis, cellular damage, or obstruction owing to crystalline precipitation. Furthermore, global reports indicate that the continued use of nonprescription nonsteroidal anti-inflammatory drugs has demonstrated allergic tubulointerstitial nephritis, and this may accelerate the progression of CKD.^24^, ^25^, ^26^

Furthermore, there are reports of other nephrotoxic drugs that compromise renal perfusion and contribute to toxicity, such as chemotherapeutic agents (mitomycin-C and immunosuppressants including cyclosporine and tacrolimus), as well as other drugs such as quinine, gemcitabine, and antiangiogenic agents. They induce thrombotic microangiopathy, impair glomerular filtration, and cause both acute and chronic kidney injury. Therefore, nephroprotective measures, safely managing drug effects on the kidneys, and using the less-toxic alternatives are essential efforts to reduce this risk.^23^^,^^27^

In addition, preventive strategies should be implemented, such as adjusting the therapeutic dose according to kidney function, ensuring continuous hydration, and regularly measuring kidney function with tests such as serum creatinine and estimated glomerular filtration rate, which is an optimal solution.^26^^,^^28^

Last but not least, new research has shown that lifestyle behaviors, obesity, low fitness levels, alcohol consumption, and a higher sugar intake are contributing indirectly to kidney dysfunction and easy impairment in a hot climate.^29^

## Literature on Heat Stress–Induced Kidney Injury in Low-Income Countries

Globally, heat stress is becoming a significant environmental and occupational risk factor for kidney health. There is much evidence and studies from around the world, especially in developing countries, about the extent of the severe damage caused by the impact of occupational heat stress on kidney health, to the point of kidney failure and death. Evidence from a study including salt pan workers in India shows a direct relation between extreme heat exposure and kidney damage, in which frequent dehydration contributes to kidney failure.^30^

Similarly, a 2016 study investigated the prevalence of indoor air pollution and its impact on kitchen workers in Lucknow, North India. The study included 95 kitchen workers and detected a high rate of kidney disease among them, suggesting significant occupational health risk associated with prolonged exposure to environmental stressors such as polycyclic aromatic hydrocarbons of a commercial kitchen.^31^^,^^32^

Research on the outbreak of CKD of undetermined origin among construction workers and farmers has been conducted in Central America, with studies highlighting the role of social, environmental, and occupational factors.^33^

Pathophysiology includes recurrent dehydration, an impaired thermoregulation system, and nephrotoxic drug exposure, all of which hasten kidney damage.^34^ Research explains the role of climate change in exacerbating the risk of kidney failure by intensifying global heat waves, thereby putting vulnerable populations at greater risk.^28^

These results underscore the crucial need for climate adaptation policies, hydration guidelines, and preventive occupational health interventions to safeguard kidney health in high-risk regions.

## Climate Change as a Health Determinant

As global average temperatures increase, so too does the frequency and severity of heat waves. The Intergovernmental Panel on Climate Change predicts a 1.5- to 2-°C increase in global temperatures by 2040, with disproportionate effects in low- and middle-income countries.^5^ In such contexts, health systems already overburdened by infectious and noncommunicable diseases may struggle to address the added load of heat-related kidney failure.

Climate change acts not only as a direct physiologic stressor but also as a social determinant of kidney health. Socioeconomic disparities exacerbate vulnerability due to unequal access to health care, cooling infrastructure, and public education on heat risk.^9^ Without urgent mitigation strategies, the incidence of heat-related kidney injury is expected to escalate.

## Potential Strategies to Mitigate Risk

### Public Health and Clinical Preparedness

An integrated response to heat-related kidney injury requires collaboration across disciplines. Public health agencies should prioritize awareness campaigns focused on hydration, heat illness symptoms, and protective behavior. Heat action plans—incorporating early warning systems, cooling centers, and occupational safeguards—have shown promise in reducing morbidity and mortality during heat waves.^5^

Clinicians, particularly nephrologists and emergency providers, must receive training to recognize and manage heat-related AKI by monitoring the temperature early, testing kidney function, and applying suitable treatment to reduce the likelihood of permanent kidney failure.^16^

In addition, as the nephrotoxic medications are a significant concern in the health care field, physicians themselves may unintentionally contribute to the spread of these drugs. Therefore, the medical team, including doctors, pharmacists, and nurses, must be made aware of the seriousness of these medications and monitor early signs of kidney damage through laboratory tests or clinical signs to ensure patient safety.^28^ Furthermore, educating patients about the risks of misuse of nonprescription medications, such as nonsteroidal anti-inflammatory drugs, and their danger to kidney health helps reduce the risk.^28^^,^^34^

Finally, we urgently call for a multipronged strategy to combat this public health crisis. Holding employers and policymakers accountable for upholding workers’ rights and dignity by ensuring safe working environments and rigorously enforcing laws against violations of occupational health and safety policies. This includes implementing simple, low-cost solutions, such as regulated rest periods, mandatory access to safe water and sanitation, and educational initiatives. Providing health insurance for periodic examinations and treatment of patients is one of the most basic rights of workers. These preventive strategies and legal legislation protect vulnerable groups, providing them with formal protection to ensure their health and safety.^30^

### Future Directions in Research and Policy

There remains a critical need for research to identify early biomarkers of heat-induced AKI and develop algorithms using artificial intelligence to assist in early laboratory detection.^35^ In addition, to gain a deeper understanding of the renal recovery trajectory and evaluate the effectiveness of community-level interventions, longitudinal studies assessing the progression from AKI to CKD in heat-exposed populations are urgently needed.

Detecting new nephrotoxic drugs on the market^35^ and investing in climate-resilient health care infrastructure, particularly in vulnerable regions, are crucial for maintaining kidney health in a warming world.

Likewise, national and international kidney disease registries should consider incorporating environmental exposure data to facilitate epidemiological tracking and targeted interventions.

Furthermore, urban planning should prioritize green infrastructure around the world to mitigate the urban heat island effect.^3^ Especially in developing countries, we know that the economies of major countries are built on the labor force of those countries. Therefore, it is the duty of international action to cooperate in establishing sustainable cities in developing countries, thereby mitigating global warming and supporting those communities in adapting to climate change.

## Conclusion

In this report, we present evidence and proof of a strong relationship between the development of CKD and occupational heat stress, particularly in vulnerable groups. The issue constitutes a “silent epidemic,” affecting a diverse range of workers and exacerbated by systemic deficiencies in labor standards and infrastructure. Therefore, research sectors and various institutions must cooperate with decision-makers in implementing future strategies that support the issue, as effective advocacy, combined with health care providers’ efforts, may help address the problem.

## References

[1] Danzl D.F., Longo D., Fauci A., Kasper D., Hauser S., Jameson J.L., Loscalzo J., Holland S., Langford C. (2026). Harrison’s Principles of Internal Medicine.

[2] Leyk D., Hoitz J., Becker C., Glitz K.J., Nestler K., Piekarski C. (2019). Health risks and interventions in exertional heat stress. Dtsch Ärztebl Int.

[3] Koh A.S.H. (2024). A cross-sectional study of heat stress exposure, heat-related symptoms, and kidney health among kitchen workers in Kampar, Perak, Malaysia.

[4] What is heat stress and how can you measure it?. https://kestrelinstruments.com/blog/what-is-heat-stress-and-how-can-you-measure-it.

[5] Casa D.J., DeMartini J.K., Bergeron M.F. (2017). National Athletic Trainers’ Association position statement: exertional heat illnesses. J Athl Train.

[6] Wen B., Xu R., Wu Y. (2021). Association between ambient temperature and hospitalization for renal diseases in Brazil during 2000-2015: a nationwide case-crossover study. Lancet Reg Health Am.

[7] Pradhan B., Kjellstrom T., Atar D. (2019). Heat stress impacts on cardiac mortality in Nepali migrant workers in Qatar. Cardiology.

[8] Tseng M.F., Chou C.L., Chung C.H. (2020). Risk of chronic kidney disease in patients with heat injury: a nationwide longitudinal cohort study in Taiwan. PLOS One.

[9] García-Arroyo F.E., Tapia E., Blas-Marron M.G. (2017). Vasopressin mediates the renal damage induced by limited fructose rehydration in recurrently dehydrated rats. Int J Biol Sci.

[10] Wang J.C., Chien W.C., Chu P., Chung C.H., Lin C.Y., Tsai S.H. (2019). The association between heat stroke and subsequent cardiovascular diseases. PLOS One.

[11] Wen F.L., Xu Y.J., Xue L.E. (2023). Proteomics analyses of acute kidney injury biomarkers in a rat exertional heat stroke model. Front Physiol.

[12] Hansson E., Glaser J., Jakobsson K. (2020). Pathophysiological mechanisms by which heat stress potentially induces kidney inflammation and chronic kidney disease in sugarcane workers. Nutrients.

[13] Nerbass F.B., Pecoits-Filho R., Clark W.F., Sontrop J.M., McIntyre C.W., Moist L. (2017). Occupational heat stress and kidney health: from farms to factories. Kidney Int Rep.

[14] Chapman C.L., Hess H.W., Lucas R.A.I. (2021). Occupational heat exposure and the risk of chronic kidney disease of nontraditional origin in the United States. Am J Physiol Regul Integr Comp Physiol.

[15] Hahn K., Kanbay M., Lanaspa M.A., Johnson R.J., Ejaz A.A. (2017). Serum uric acid and acute kidney injury: a mini review. J Adv Res.

[16] Périard J.D., Eijsvogels T.M.H., Daanen H.A.M. (2021). Exercise under heat stress: thermoregulation, hydration, performance implications, and mitigation strategies. Physiol Rev.

[17] Pal J.S., Eltahir E.A.B. (2016). Future temperature in southwest Asia projected to exceed a threshold for human adaptability. Nat Clim Change.

[18] Kenny G.P., Sigal R.J., McGinn R. (2016). Body temperature regulation in diabetes. Temperature.

[19] Wesseling C., Aragon A., Gonzalez M. (2016). Kidney function in sugarcane cutters in Nicaragua–a longitudinal study of workers at risk of Mesoamerican nephropathy. Environ Res.

[20] Shubham S., Kumar M., Sarma D.K. (2022). Role of air pollution in chronic kidney disease: an update on evidence, mechanisms and mitigation strategies. Int Arch Occup Environ Health.

[21] Afsar B., Elsurer Afsar R., Kanbay A., Covic A., Ortiz A., Kanbay M. (2019). Air pollution and kidney disease: review of current evidence. Clin Kidney J.

[22] Kakamu T., Endo S., Hidaka T., Masuishi Y., Kasuga H., Fukushima T. (2021). Heat-related illness risk and associated personal and environmental factors of construction workers during work in summer. Sci Rep.

[23] Patel J.B., Sapra A. (2025). http://www.ncbi.nlm.nih.gov/books/NBK553144/.

[24] Farquhar W.B., Morgan A.L., Zambraski E.J., Kenney W.L. (1999). Effects of acetaminophen and ibuprofen on renal function in the stressed kidney. J Appl Physiol.

[25] Glaser J., Lemery J., Rajagopalan B. (2016). Climate change and the emergent epidemic of CKD from heat stress in rural communities: the case for heat stress nephropathy. Clin J Am Soc Nephrol.

[26] Kwiatkowska E., Domański L., Dziedziejko V., Kajdy A., Stefańska K., Kwiatkowski S. (2021). The mechanism of drug nephrotoxicity and the methods for preventing kidney damage. Int J Mol Sci.

[27] Mitchel J. (2023). Nephroprotective measures and safely managing drug effects on kidneys. J Pharmacol Rep.

[28] Sasai F., Roncal-Jimenez C., Rogers K. (2023). Climate change and nephrology. Nephrol Dial Transplant.

[29] Li M., Shu W., Amaerjiang N. (2022). Interaction of hydration status and physical activity level on early renal damage in children: a longitudinal study. Front Nutr.

[30] Venugopal V., Lennqvist R., Latha P.K. (2023). Occupational heat stress and kidney health in salt pan workers. Kidney Int Rep.

[31] Singh A., Kamal R., Mudiam M.K. (2016). Heat and PAHs emissions in indoor kitchen air and its impact on kidney dysfunctions among kitchen workers in Lucknow, North India. PLOS One.

[32] Saif Eldin A.S., Radwan N.A., Khalifa E.M. (2022). Evaluation of occupational indoor heat stress impact on health and kidney functions among kitchen workers. Egypt J Occup Med.

[33] Jayasumana C., Orantes C., Herrera R. (2017). Chronic interstitial nephritis in agricultural communities: a worldwide epidemic with social, occupational and environmental determinants. Nephrol Dial Transplant.

[34] Chapman C.L., Johnson B.D., Parker M.D., Hostler D., Pryor R.R., Schlader Z. (2020). Kidney physiology and pathophysiology during heat stress and the modification by exercise, dehydration, heat acclimation and aging. Temperature.

[35] Džidić-Krivić A., Sher E.K., Kusturica J., Farhat E.K., Nawaz A., Sher F. (2024). Unveiling drug induced nephrotoxicity using novel biomarkers and cutting-edge preventive strategies. Chem Biol Interact.

